# Efficient three-dimensional point cloud object detection based on improved Complex-YOLO

**DOI:** 10.3389/fnbot.2023.1092564

**Published:** 2023-02-16

**Authors:** Yongxin Shao, Zhetao Sun, Aihong Tan, Tianhong Yan

**Affiliations:** College of Mechanical and Electrical Engineering, China Jiliang University, Hangzhou, China

**Keywords:** deep learning, object detection, point cloud, 3D object detection, neural network

## Abstract

Lidar-based 3D object detection and classification is a critical task for autonomous driving. However, inferencing from exceedingly sparse 3D data in real-time is a formidable challenge. Complex-YOLO solves the problem of point cloud disorder and sparsity by projecting it onto the bird’s-eye view and realizes real-time 3D object detection based on LiDAR. However, Complex-YOLO has no object height detection, a shallow network depth, and poor small-size object detection accuracy. To address these issues, this paper has made the following improvements: (1) adds a multi-scale feature fusion network to improve the algorithm’s capability to detect small-size objects; (2) uses a more advanced RepVGG as the backbone network to improve network depth and overall detection performance; and (3) adds an effective height detector to the network to improve the height detection. Through experiments, we found that our algorithm’s accuracy achieved good performance on the KITTI dataset, while the detection speed and memory usage were very superior, 48FPS on RTX3070Ti and 20FPS on GTX1060, with a memory usage of 841Mib.

## 1. Introduction

Deep learning-related technologies are increasingly integrated into people’s daily life, and object detection algorithms (Qi et al., 2021; Liu et al., 2022a,b; Xu et al., 2022), as a crucial component of the autonomous driving perception layer, can create a solid foundation for behavioral judgments during autonomous driving. Although object detection algorithms based on 2D images (Bochkovskiy et al., 2020; Bai et al., 2022; Cheon et al., 2022; Gromada et al., 2022; Long et al., 2022; Otgonbold et al., 2022; Wahab et al., 2022; Wang et al., 2022) have had a lot of success at this stage, single-view images cannot completely reflect the position pose, and motion orientation of objects in 3D space due to the lack of depth information in 2D images. Consequently, in the field of autonomous driving, the focus of object detection research has increasingly switched from 2D image detection to 3D image detection and point cloud detection. To compensate for the lack of depth information in 2D images, researchers primarily use multi-view (Khatab et al., 2022; Siddique and Lee, 2022) and depth cameras (Perek et al., 2022) when studying image-based 3D object detection algorithms. However, because both depth cameras and ordinary 2D cameras are affected by light, image-based object detection algorithms frequently perform poorly in complex environmental conditions. Compared with images, point cloud data collected by LIDAR not only provides accurate geometric features but also has better robustness to light. However, point cloud-based 3D object detection algorithms face significant challenges. For instance, (1) Sparsity: Compared with the dense pixel arrangement in images, point cloud data are sparse and unevenly distributed; (2) Disorder: The nature of the object is unaffected by the ordering of the point cloud data; and (3) Rotation invariance: The coordinates of all points in a point cloud collection change when rotated, yet they still represent the same object (Qi et al., 2017a). To resolve these issues Simony et al. (2018) proposed Complex-YOLO to complete 3D object detection by projecting 3D point cloud data onto the 2D bird’s eye view, transforming the disordered point cloud into the 2D pseudo-image after meshing it and using the image-based 2D object detection algorithm. Complex-YOLO uses YOLOV2 (Redmon and Farhadi, 2017) for feature extraction, classification, and regression, and extends the E-RPN (Euler Region Proposal Network) structure for object orientation angle regression. However, because Complex-YOLO lacks a neck network, it must complete object detection at a lower resolution, resulting in poor performance for small objects. It performs poorly in 3D bounding box regression because it does not complete the detection of object height and instead provides a fixed height value for different classes of objects. In this paper, the proposed Efficient Complex-YOLO (from now on, we will call our proposal as Efficient Complex-YOLO) improves Complex-YOLO comprehensively, chooses a more advanced network structure, makes up for the original lack of Complex-YOLO for height detection, and substantially improves the detection accuracy while ensuring real-time detection, the main work is as follows:

(1) A more advanced 2D object detection technique with a more efficient feature extraction network [RepVGG-A2 (Ding et al., 2021)] is used.
(2) Building an improved FPN [Feature Pyramid Network (Lin et al., 2017a)] structure to accomplish the detection task at different resolutions, which improves the detection accuracy for small objects.
(3) A more efficient head detection network is designed to complete the detection of object height, cancel the E-RPN structure, and complete the detection of object orientation angle directly in the Cartesian coordinate system to achieve true 3D object detection.

The rest of this paper is as follows.

(1) Section 2 presents the work related to the point cloud-based 3D object detection algorithm.
(2) Section 3 presents the algorithmic structure of Efficient Complex-YOLO.
(3) Section 4 will experimentally verify the effectiveness of the improvements made and compares them with other point cloud-based 3D object detection algorithms.
(4) Section 5 concludes the whole paper.

## 2. Related work

The point cloud-based 3D object detection algorithms can be divided into three categories based on the pre-processing techniques used on point cloud data. (1) 3D object detection with Point-based methods; (2) 3D object detection with Voxel-based methods; and (3) 3D object detection with Grid-based methods.

### 2.1. 3D object detection with point-based methods

The PointNet proposed by Qi et al. (2017a) processes point cloud data directly. PointNet solves the point cloud rotation invariance problem using a spatial transformation network, solves the point cloud disorder problem using the Max-pooling method, and builds a unified system framework for object classification and semantic segmentation, and many subsequent algorithms will use this network to extract features. However, PointNet can only characterize each point and cannot effectively integrate the point cloud information of a region of the point cloud. The subsequently proposed PointNet++ (Qi et al., 2017b) effectively solves this problem by drawing on the idea of hierarchical feature extraction; it consists of three segments: sampling, grouping, and feature extraction, using the farthest point sampling method to extract key points from the original point cloud for sampling, then using PointNet to extract features from the grouped point set to select key point features for subsequent semantic segmentation or 3D object detection. The PointRCNN algorithm proposed by Shi et al. (2019) is the first 3D object detection method that is entirely based on point clouds; it is a two-stage object detection algorithm that uses PointNet as the feature extraction network. The first stage performs binary classification of point clouds, classifying each point into the foreground or background, and generating a bounding box for each foreground point; the second stage optimizes the bounding box generated in the first stage, and the features of each bounding box are obtained through point cloud pooling and other operations; finally, the bounding box is optimized and scored with confidence by combining the features obtained in the first stage, to obtain the final bounding box. However, unlike object detection on 2D images, point cloud data do not have a regular grid, making it difficult to apply the idea of 2D detection to complete the regression of centroids and bounding boxes for objects whose centroids are outside the point cloud. The VoteNet algorithm proposed by Qi et al. (2019) uses the Hoff voting mechanism to continuously generate some virtual points close to the object’s centroid, and then completes the classification and regression tasks using a clustering algorithm and some classifiers and regressors. Many point-based 3D object detection algorithms currently use hand-crafted grouping schemes to group point clouds and extract local features using feature extraction networks like PointNet. However, the hand-crafted grouping scheme leads to inaccurate point assignment and degrades the performance of 3D object detection. The Group-Free 3D algorithm proposed by Liu et al. (2021) uses Transformer (Vaswani et al., 2017) to adaptively determine which points are effective for the current object detection; it uses Transformer’s attention mechanism to compute the object features of all points in the point cloud and automatically learns the contribution of each point during network training. An improved attention stacking scheme is then used to fuse object features from different stages to produce more accurate object detection results.

### 2.2. 3D object detection with voxel-based methods

A voxel is similar to a pixel in an image. The smallest unit in an RGB image is the pixel, while the smallest unit in a three-dimensional spatial representation is the voxel. If the whole space is divided into small cubes into three dimensions, each small cube is a voxel. The voxelization process is to use voxels to express the appearance and shape of an object at the corresponding position in the three-dimensional space, and by doing so, the disordered point cloud can be sorted and the internal information of the object can be well described. VoxelNet proposed by Zhou and Tuzel (2018) divides the 3D point cloud into a certain number of voxels, and then, after random sampling of points and normalization, for each non-empty voxel use VFE (Voxel Feature Encoding) layers for local feature extraction to obtain Voxel-wise feature. Then 3D Convolution Middle Layers are used to further abstract the features (increase the perceptual field and learn the geometric space representation), and RPN (Region Proposal Network) is used for object classification and regression. However, 3D convolution tends to consume a lot of computing power. The subsequent SECOND proposed by Yan et al. (2018) makes a series of improvements based on VoxelNet and proposes an improved sparse convolution method that increases the detection speed, reduces the memory usage, and improves the orientation angle detection performance by introducing an angle loss method. Lang et al. (2019) subsequently proposed PointPillars, which retains the idea of VoxelNet and Second, but unlike the former, divides the point cloud into individual pillars for feature extraction and replaces the original 3D convolution with 2D convolution. Shi et al. (2020a) proposed a new 3D detection network called PV-RCNN, which improves the detection accuracy by combining two different data forms, point clouds, and voxels, and extracting features from them. The algorithm combines the efficient feature extraction capability of 3D convolution with the advantage of point clouds’ variable perceptual field.

### 2.3. 3D object detection with grid-based methods

These algorithms usually project point cloud data into the 2D grid, then use 2D convolution for feature extraction. Chen et al. (2017) proposed MV3D to achieve 3D object detection by multi-view fusion, firstly computing candidate regions on the bird’s-eye view of the point cloud, then integrating the candidate regions with point cloud bird’s-eye view features, point cloud front view features, and image features into one dimension, respectively by Roi (Region of Interest) pooling, and finally using convolution neural networks to complete classification and regression. Subsequently, Ku et al. (2018) proposed AVOD based on MV3D by eliminating the input of the point cloud main view and adding intensity features in the point cloud bird’s-eye view, followed by modifying the feature extraction network to improve small object detection accuracy.

## 3. Materials and methods

### 3.1. Detection process

**FIGURE 1:**
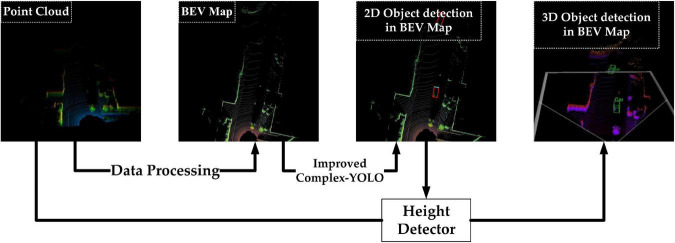
3D object detection flow chart.

The overall detection algorithm is shown in Figure 1 above, which has the following four steps:

Step 1 Data pre-processing. Rasterized projection of point cloud data in the bird’s-eye view to generate the 2D pseudo-image (from now on, we will call it as BEV-map);
Step 2 BEV-map detection. The Bev-map is fed into the improved Complex YOLO to complete the extraction of feature maps and the detection of the 2D bounding box in the bird’s eye view and the detection of object orientation angle;
Step 3 Height detection. The feature maps obtained in step 2 are fed into the height detector to output the object’s height;
Step 4 Finally. The information obtained from step 2 is fused with step 3 to complete the 3D object detection task;

### 3.2. Data preprocessing

In this paper, we generate BEV-map using the same data pre-processing method as Complex-YOLO by rasterizing and projecting the single-frame point cloud data covering the 50 m × 50 m area in front onto the bird’s-eye view map, and the grid size of BEV-map is *m* = 608 and *n* = 608, corresponding to the size of each grid is about *g* = 0.8. Then the maximum height, maximum intensity, and density of the point cloud data in the bird’s-eye view are encoded and filled into the RGB triple channel. Considering that the height of LIDAR is about 1.73 m during data acquisition, while most targets are between 0 and 4 m high, to avoid the influence of obscurants such as trees, we selected the point cloud data in the range of *z* ∈ [2.73, 1.27]. All point clouds were defined *P*_Ω_ as:


(1) $$P_{\Omega }=\{P={\left[x,y,z\right]}^{T}|x\in \left[0,50m\right],y\in \left[25,25m\right],z\in \left[2.73,1.27m\right]\}$$

The three channels of the transformed image are encoded by the following equations (2), (3), and (4) for point cloud height information, point cloud intensity information, and point cloud density information, respectively.


(2) $$z_{g}=max\left(P_{\Omega \text{i}\to \text{j}}\cdot {\left[0,0,1\right]}^{T}\right)$$

(3) $$z_{b}=max\left(I\left(P_{\Omega \text{i}\to \text{j}}\right)\right)$$

(4) $$z_{r}=min\left(1.0,log\left(N+1\right)/log\left(64\right)\right)$$

In above equations (2), (3), and (4) above, **I**(*P*_Ω_) represents Point cloud intensity; *N* represents the number of point clouds in each grid; **z_g_** represents the maximum height; **z_b_** represents the maximum intensity; **z_r_** the normalized density within the grid; **z_b_**, **z**_**g**,_ and **z_r_** the final output RGB-map pixel values for each channel.

### 3.3. Complex-YOLO and its improved parts

**FIGURE 2:**
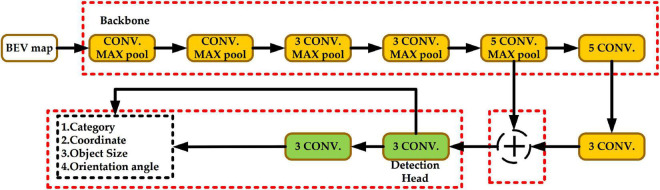
Complex-YOLO network structure.

Figure 2 shows the network structure of Complex-YOLO. The yellow block represents the backbone network, the green block represents the head network, and the red dashed box is the part to be improved in this paper.

**FIGURE 3:**
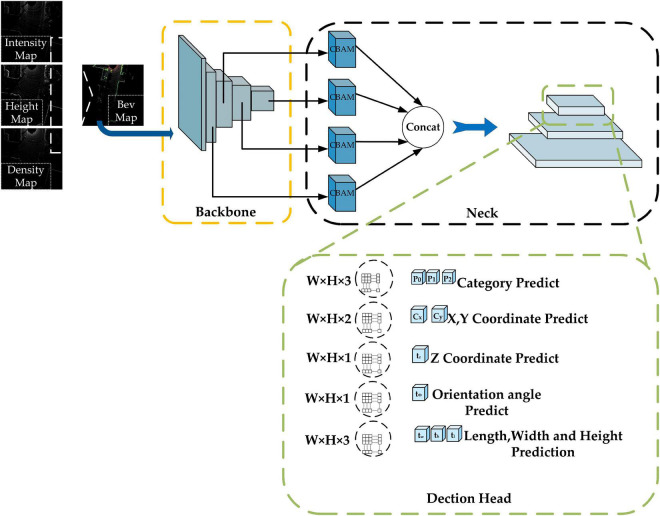
Efficient Complex-YOLO network structure. The yellow-black-green dashed boxes represent the Backbone, Neck, and Head parts of the network, respectively. W, H, and C represent the length, width, and the number of channels of the feature map, respectively. Efficient Com-plex-YOLO detects small and large objects at 152 × 152, 76 × 76, and 38 × 38 resolutions, respectively.

In this paper, a comprehensive improvement is made to Complex-YOLO, and the Efficient Complex-YOLO network structure is shown in Figure 3, as follows.

(1) Improvement of the backbone network part: using RepVGG as the backbone network to extract feature maps.
(2) Adding the neck network structure: adding FPN+CBAM (Convolutional Block Attention Module) (Woo et al., 2018) as the neck network to achieve special multi-scale fusion.
(3) Improvements in the head network: selection of the Anchor-free approach, in addition to removing the E-RPN structure in the head network and adding an efficient height detector to regress height.

#### 3.3.1. Backbone network improvements

**FIGURE 4:**
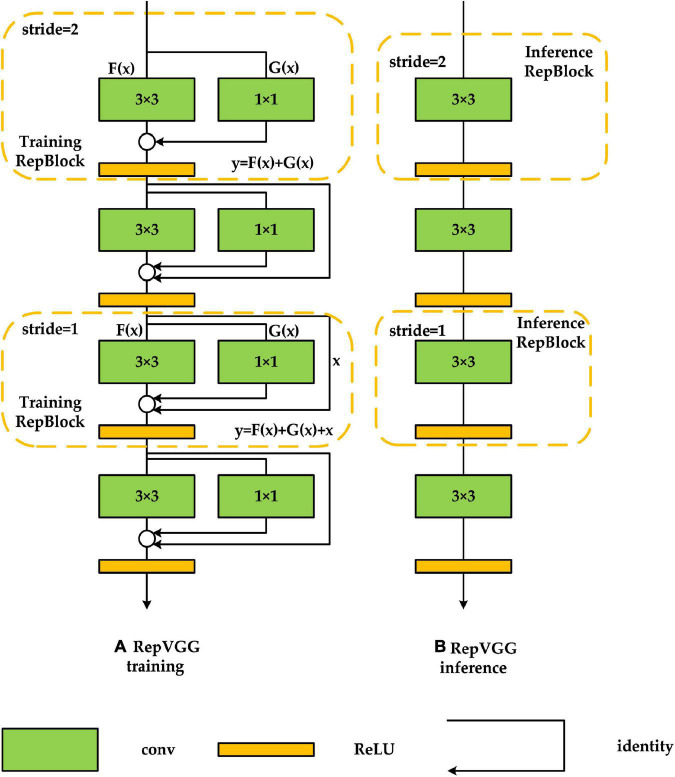
RepVGG network structure. **(A,B)** Are the network structures of RepVGG in the training and inference process, respectively.

To meet the demand for real-time detection while maintaining detection accuracy, we used RepVGG-A2 in the feature extraction network section, which performs better in terms of detection accuracy, detection speed, and memory usage, and the specific structure is shown in Figure 4. RepVGG is a feature extraction network proposed by Ding et al. (2021). Compared with other multi-branch structures [e.g., ResNet (He et al., 2016), DenseNet (Huang et al., 2017)], RepVGG converts the multi-branch structure into a single-branch structure consisting entirely of 3 × 3 convolutions in the inference stage, which can improve the detection speed of the algorithm and reduce the memory usage while maintaining a high detection accuracy. Figure 4A shows the RepVGG training phase structure. In the training phase, two branching structures are used: one is a residual structure similar to ResNet with *y* = *x* + *F*(*x*), and the other is a branching structure with *y* = *G*(*x*) + *F*(*x*), to which a 1 × 1 convolution is added for feature extraction, and the final performance is *y* = *x* + *F*(*x*) + *G*(*x*). This branching structure provides the network with numerous gradient update pathways, making the trained model more adaptable. Training such a network is similar to the concept of model integration, in which many models are fused to improve model robustness and reduce gradient disappearance, which is more favorable to loss function convergence. Figure 4B shows the network structure of RepVGG during the inference phase. In the inference phase, RepVGG reconstructs the model weights of the identity branch and 1 × 1 branch into 3 × 3 convolution, and then fuses the reconstructed parameters with the original 3 × 3 convolution weights of the main branch, resulting in a network consisting solely of 3 × 3 convolution. This method can avoid the need to save the calculation results of all branches before completing the feature fusion operation of the residual structure during the actual program operation and effectively reduce memory usage while ensuring accuracy and greatly improving detection speed. In addition, most algorithmic frameworks nowadays accelerate 3 × 3 convolution, and with GPU acceleration, the FLOPS (computational density, number of floating points operations per second) of 3 × 3 convolution can reach four times that of 1 × 1 convolution and 5 × 5 convolution, which greatly accelerates RepVGG’s detection speed (Ding et al., 2021).

The model used in this paper is the RepVGG-A2 model. The specific parameters are shown in Table 1.

**TABLE 1:** RepVGG-A2 specific parameters.

Stage	Output size	Number of blocks
1	304 × 304 × 64	1
2	152 × 152 × 69	1
3	76 × 76 × 192	2
4	38 × 38 × 384	14
5	19 × 19 × 1,408	1

#### 3.3.2. Neck network improvements

There is no neck network in the original Complex-YOLO. However, when the convolution neural network is used for feature extraction, the size of the extracted feature maps decreases steadily as the network level increases, and the perceptual field corresponding to the regions in the original image will gradually get larger, resulting in the loss of feature information for small objects and making small object detection difficult. Based on this, this paper adds a neck network to the original Complex-YOLO and uses the structure of FPN+CBAM to improve the detection of small-sized objects.

**FIGURE 5:**
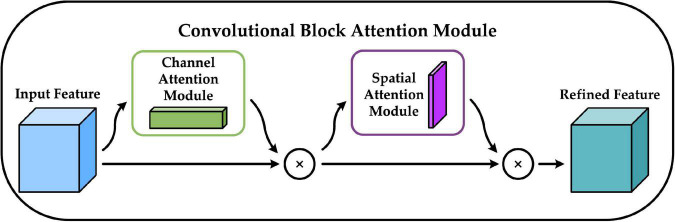
Convolutional block attention module (CBAM) network structure.

We add the CBAM module before constructing the FPN structure. CBAM is a convolutional block attention module proposed by Woo et al. (2018) that combines the channel attention mechanism and the spatial attention mechanism, and the specific structure is shown in Figure 5. The channel attention mechanism generates a channel attention map by exploiting the inter-channel relationships of features. As each channel of a feature map is considered as a feature detector, channel attention focuses on “what” is meaningful given an input image. The spatial attention mechanism uses the spatial relationships of features to build a spatial attention map. It focuses on “where” is an informative part, which is complementary to the channel attention. Adding the CBAM module before building the FPN structure enables the model to learn which features are more beneficial to the detection task, eliminates redundant features, and facilitates the integration of different levels of semantic information in the FPN structure (Lin et al., 2017a).

**FIGURE 6:**
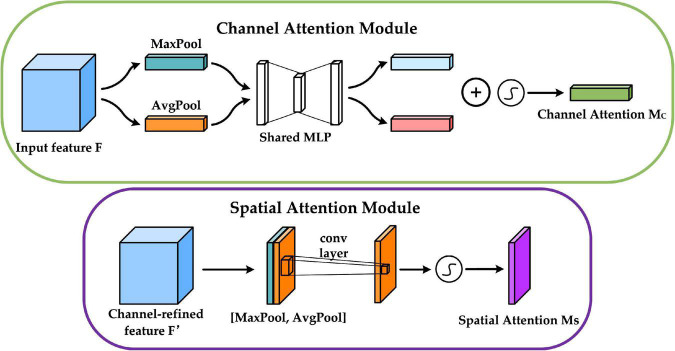
Structure of channel attention module and spatial attention module.

The upper part of Figure 6 shows the channel attention mechanism, and its implementation can be divided into two parts that will execute global average pooling and global max pooling for each of the individual feature layers coming in. Following that, the results of the average pooling and max pooling are processed using a shared fully connected layer, and the two processed results are summed, followed by a Sigmoid operation to produce the weights (between 0 and 1) of each channel of the input feature layer. After obtaining this weight, the spatial attention map is generated by multiplying this weight by the original input feature layer. The bottom half of Figure 6 shows the spatial attention mechanism, which takes the maximum value and average value on each channel for each feature point, for the input to be passed into the feature layer. Following that, a stacking of these two results is performed, and the number of channels is adjusted using a convolution with one channel at a time, and then the same Sigmoid operation is conducted to acquire the weight (between 0 and 1) of each feature point of the input feature layer, and finally, this weight is multiplied by the original input feature layer to generate the channel attention map.

The input features are defined as *F* ∈ R^*C* × *W* × *H*^, *M*_*C*_ ∈ R^*C* × 1 × 1^,and *M*_*S*_ ∈ *R*^1 × *W* × *H*^, which are CBAM-derived one-dimensional channel attention weights and two-dimensional spatial attention weights. Then *M_C_* and *M_S_* are described by equations (5) and (6).


(5) $$\begin{array}{l} M_{C}=\sigma \left(MLP\left(AvgPool\left(F\right)\right)+MLP\left(MaxPool\left(F\right)\right)\right)\\ =\sigma (W_{1}\left(W_{0}\left(F_{avg}^{C}\right)\right)+W_{1}(W_{0}(F_{max}^{C}))) \end{array}$$

(6) $$\begin{array}{l} M_{S}=\sigma \left(f^{7\times 7}\left(\left[AvgPool\left(F\right);;MaxPool\left(F\right)\right]\right)\right)\\ =\sigma (f^{7\times 7}(\left[F_{avg}^{S};;F_{max}^{S}\right])) \end{array}$$

Where σ denotes the Sigmoid activation function; *f*^7 × 7^ denotes the 7 × 7 convolution operation; *W*_0_ ∈ R^*C*/*r* × *C*^, *W*_1_ ∈ R^*C* × *C*/*r*^ denote the weight parameters of the MLP (Multi-Layer Perception), where *r* is the reduction ratio; $F_{avg}^{C}$, $F_{max}^{C}$ denote the average pooled and maximum pooled features; $F_{avg}^{S}$, $F_{max}^{S}$ denote the average pooled and max pooled features across channels.

Equations (7) and (8) define the computational process of CBAM by defining *F* as the output of the channel attention mechanism and *F* as the output of the spatial attention mechanism. ⊗ denotes element-wise multiplication.


(7) $$F=M_{C}\left(F\right)⊗F$$

(8) $$F=M_{C}\left(F\right)⊗F$$

**FIGURE 7:**
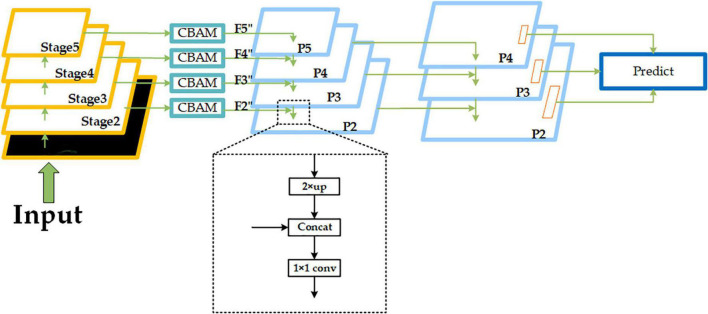
Improved feature pyramid network (FPN) network structure. The “Concat” module means stitching by channel dimension. The dotted box part shows one feature fusion operation of adjacent feature maps.

As shown in Figure 7, we select the feature maps output from Stage2, Stage3, Stage4, and Stage5 of RepVGG-A2 (corresponding resolutions 152 × 152, 76 × 76, 38 × 38, and 19 × 19, in that order) as input, and then build the FPN structure after passing through the CBAM module. The outputs through CBAM are defined as F2, F3, F4, and F5, and we will do 2× up-sampling of the lower resolution feature map in the adjacent feature maps, then stitch it with the adjacent feature maps of the higher resolution by channel dimension (for example, the resolution of F5 is 19 × 19, and the resolution of F4 is 38 × 38, so we do 2× up-sampling of F5, and then stitch it with F4 by channel dimension), and finally, adjust the number of channels by 1 × 1 convolution. This completes the feature fusion of adjacent feature maps. The fused results are defined as P2, P3, P4, and P5. In the course of subsequent experiments, we found that when predicted at a resolution of 152 × 152, each grid corresponds to a size of about 0.32 m × 0.32 m in the original 50 m × 50 m space, which is not conducive to pedestrian detection in dense scenes, so we subjected the obtained P2, P3, and P4 to another 2× up-sampling operation and completed a feature of adjacent resolution fusion, and the final set of feature maps participating in the prediction is defined as (P2, P3, P4, corresponding resolutions 304 × 304, 152 × 152, and 76 × 76). In general, the lower-resolution feature map contains more semantic information, and each grid has a wider perceptual range, whereas the higher-resolution feature map contains rich spatial information, and each grid has a narrower perceptual range. As a result, by constructing an FPN structure, we can fuse different feature information of different resolutions on the one hand, and predict objects of different sizes in different perceptual ranges on the other. We use P4 to predict some larger objects and P3 and P2 to predict some smaller objects during the prediction stage.

#### 3.3.3. Head network improvements

**FIGURE 8:**
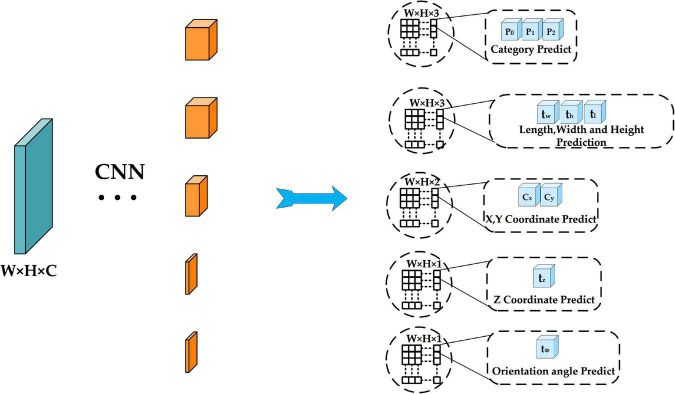
Improve head network structure.

Complex-YOLO adds the E-RPN network based on YOLOv2 to achieve the regression of object orientation angles and in the Efficient Complex-YOLO, we choose to regress the angle of the object directly. In addition, Complex-YOLO does not detect the height of the object but instead gives a fixed height value for different classes of objects (Car:1.5 m; Cyclist:1.4 m; Pedestrian:1.8 m), which is still essentially 2D object detection and is not conducive to the return of the 3D bounding box. Efficient Complex-YOLO adds the height detector based on Complex-YOLO to directly predict the height of objects, which greatly improves the detection accuracy of the 3D Bounding box. Figure 8 shows the specific head prediction network structure.

For the prediction of 2D bounding box information, we choose the Anchor-free based prediction method. The anchor proposed by Faster R-CNN (Ren et al., 2015) tries to predetermine the shape of objects for different shapes and sizes, it can handle different shapes of objects well and also reduces the difficulty of training. However, the foreground points (objects we want to detect, such as cars, pedestrians, etc.) in a point cloud data frame are few, resulting in a large number of anchor boxes matching the background points and being judged as negative samples, and only a few anchor boxes matching the foreground points and being judged as positive samples, resulting in serious positive and negative sample mismatch, which is detrimental to model training.

In the prediction stage, we predict five results for each output of the FPN structure (both P2, P3, and P4 mentioned above), corresponding to the predicted class confidence of the object, the 2D bounding box coordinate offset, the orientation angle of the object, the z-axis coordinate offset under the 3D bounding box, and the length, width, and height of the 3D bounding box. Each prediction result is completed by two convolutions. For the first time, 3 × 3 convolution is used to adjust the number of channels. For example, for category probability prediction of objects, the feature map size is integrated from W × H × C to W × H × 3 by a 3 × 3 convolution operation (we selected three categories of Car, Pedestrian and Cyclist for testing. Therefore, the channel number of the integrated feature map is 3). Finally, we use a 1 × 1 convolution operation to complete the prediction.

In Complex-YOLO, the E-RPN network decomposes the regression of the object orientation angle into the regression of the parameters *t*_*im*_ and *t*_*re*_, which correspond to the phase of a complex number. *artan*(*t*_*im*_, *t*_*re*_) is used to calculate the orientation angle (Simony et al., 2018). We attempt to predict the orientation angle of the object directly, and subsequent experiments show that direct regression of the angle can improve the algorithm’s detection accuracy even further.

As previously stated, the BEV-map constructed during the data pre-processing stage contains the maximum height information of the point cloud data in each grid, and we chose point cloud data in the range of *z* ∈ [2.73, 1.27], which can filter out the majority of the occlusion cases. As a result, we add the height detector using the same procedure as the category prediction described above (3 × 3 convolution is used to integrate the feature map size from W × H × C to W × H × 1, and then 1 × 1 convolution is used to complete the final height detection).

Let’s define *b_x_, b_y_, b_z_* as the center point coordinates of the 3D bounding box, *b_w_, b_h_, b_l_*as the length, width, and height of the 3D bounding box, *b*_ϕ_ as the orientation angle, *t_x_, t_y_, t_z_, t_w_, t_h_, t_l_, t*_ϕ_ as the relative offset obtained from the network prediction, *c_x_, c_y_* as the grid center point coordinates in the bird’s eye view, and σ(*x*) as the Sigmoid function. Equations (9) to (15) indicate the regression modes of 3D bounding boxes of objects.


(9) $$b_{x}=\sigma \left(t_{x}\right)+c_{x}$$

(10) $$b_{y}=\sigma \left(t_{y}\right)+c_{y}$$

(11) $$b_{z}=t_{z}$$

(12) $$b_{w}=t_{w}$$

(13) $$b_{h}=t_{h}$$

(14) $$b_{l}=t_{l}$$

(15) $$b_{\phi }=t_{\phi }$$

In the training phase, inspired by CenterNet (Duan et al., 2019), we choose to generate a heatmap to calculate the object category loss and Focal Loss (Lin et al., 2017b) is chosen to calculate the category loss of objects to avoid the problem of positive and negative sample mismatch. The specific calculation formula is shown in equation (16).


(16) $$L_{cls}=\frac{-1}{N}\sum_{c=1}^{C}\sum_{w=1}^{W}\sum_{h=1}^{H}\left\{ \begin{array}{cc} {\left(1-{\hat{p}}_{cij}^{cls}\right)}^{\alpha }\log\left({\hat{p}}_{cij}^{cls}\right) & ifp_{cij}^{cls}=1\\ {\left(1-p_{cij}^{cls}\right)}^{\beta }\log\left(1-{\hat{p}}_{cij}^{cls}\right) & otherwise \end{array}\right.$$

*C* denotes the number of channels of the heatmap (in this paper, we selected three categories of Car, Pedestrian and Cyclist for testing, so *C* = 3 here); *H* and *W* denotes the length and width of heatmap; $p_{cij}^{cls}$ can be obtained from the length and width of the ground truth as well as the Gaussian kernel calculated; ${\hat{p}}_{cij}^{cls}$ is the predicted value of the network; we set α to 2 and β to 4.

We used L1 Loss to calculate the loss values of the x, y coordinate offset and the loss values of the orientation angle of the object. The specific calculation formula is shown in equations (17) (18).


(17) $$L_{yaw}=\frac{1}{N}\sum_{w=1}^{W}\sum_{h=1}^{H}1_{ij}^{obj}\left|{\hat{p}}_{ij}^{yaw}-p_{ij}^{yaw}\right|$$

(18) $$L_{off}=\frac{1}{N}\sum_{w=1}^{W}\sum_{h=1}^{H}1_{ij}^{obj}\left|{\hat{p}}_{ij}^{off}-p_{ij}^{off}\right|$$

$p_{ij}^{yaw}$, ${\hat{p}}_{ij}^{yaw}$ denote the ground truth and predicted values of orientation angle; $p_{ij}^{off}$, ${\hat{p}}_{ij}^{off}$ denote the ground truth and predicted values of x, y offsets, respectively; $1_{ij}^{obj}$ denotes if an object appears in position i, j.

We use Balanced L1 Loss (Pang et al., 2019) to calculate the loss values of the z-coordinate and length, width and height, the specific formula as shown in equations (19) (20).


(19) $$\begin{array}{l} L_{dim}=\frac{1}{N}\sum_{w=1}^{W}\sum_{h=1}^{H}1_{ij}^{obj}\\ \;\{\begin{array}{c} \frac{\alpha }{b}\left(b\left|{\hat{p}}_{ij}^{dim}-p_{ij}^{dim}\right|\right)ln\left(b\left|{\hat{p}}_{ij}^{dim}-p_{ij}^{dim}\right|\right)if\left|{\hat{p}}_{ij}^{dim}-p_{ij}^{dim}\right|=1\\ \;\gamma \left|x\right|+C\;otherwise \end{array} \end{array}$$

(20) $$\begin{array}{l} L_{z}=\frac{1}{N}\sum_{w=1}^{W}\sum_{h=1}^{H}1_{ij}^{obj}\\ \;\{\begin{array}{c} \frac{\alpha }{b}\left(b\left|{\hat{p}}_{ij}^{z}-p_{ij}^{z}\right|\right)ln\left(b\left|{\hat{p}}_{ij}^{z}-p_{ij}^{z}\right|\right)if\left|{\hat{p}}_{ij}^{z}-p_{ij}^{z}\right|=1\\ \;\gamma \left|x\right|+C\;otherwise \end{array} \end{array}$$

$p_{ij}^{dim}$, ${\hat{p}}_{ij}^{dim}$ denote the ground truth and predicted values of the object’s length, width and height, respectively; $p_{ij}^{z}$, ${\hat{p}}_{ij}^{z}$ denote the ground truth and predicted values of the object’s z-coordinate; α and γ are 0.5 and 1.5, respectively; α, γ and *b* satisfy the relation *b* = *e*^γ/α^ − 1;

The final loss function is calculated as shown in equation (21).


(21) $$L_{total}=\lambda_{cls}L_{cls}+\lambda_{yaw}L_{yaw}+\lambda_{off}L_{off}+\lambda_{z}L_{z}+\lambda_{dim}L_{dim}$$

λ_*cls*_, λ_*yaw*_, λ_*off*_, λ_*z*_ and λ_*dim*_ denote the weight of each part of the loss function, respectively.

## 4. Result

### 4.1. Experiment environment

The experimental platform in this paper is Ubuntu 18.04; the GPU used is Nvidia RTX3070Ti with 8G memory; the deep learning framework is PyTorch 1.10; the KITTI (Geiger et al., 2012) dataset is selected for model training, 6,000 data are randomly selected as the training set, and the rest are used as the validation set.

### 4.2. Ablation experiment design and analysis

The experiments in this paper used the same KITTI dataset used by Complex-YOLO to evaluate detection accuracy for three categories of objects: Car, Pedestrian, and Cyclist. AP (Average Precision) and mAP (Mean Average Precision) are used as accuracy evaluation metrics, while FPS (Frames Per Second) is used as speed evaluation metrics. The IoU (Intersection over Union) threshold is 0.7 for Car and 0.5 for Pedestrian and Cyclist (for example, if the IOU threshold is 0.7, when the IOU between the detected bounding box and the ground truth bounding box is greater than 0.7, the detected bounding box is considered to be a positive sample), and detection results are classified as Easy, Mod, and Hard depending on the object being obscured. Tables 2, 3 show the specific experimental results, where Table 2 shows the accuracy of 2D object detection in the bird’s eye view perspective and Table 3 shows the accuracy of 3D object detection.

**TABLE 2:** Experimental results of the ablation experiments were used to verify the contribution of the improvements in this paper.

Improved part		Method A	Method B	Method C	Method D	Method E	Method F	Method G
E-RPN		√	√	√	√			
RepVGG-A2			√	√	√	√	√	√
FPN				√	√	√	√	√
Height detector					√	√	√	√
CBAM							√	√
Higher resolution								√
Car AP	Easy	85.89	89.46	84.79	92.73	87.96	**97.34**	96.52
	Mod	77.40	89.36	87.18	87.20	88.24	89.74	**89.80**
(IoU = 0.7)	Hard	77.33	80.80	87.81	87.83	88.61	89.90	**89.92**
Pedestrian AP	Easy	46.08	36.96	66.91	66.76	67.94	71.88	**73.26**
	Mod	45.90	32.88	69.27	69.27	70.51	73.44	**73.56**
(IoU = 0.5)	Hard	44.20	33.84	70.63	70.63	71.35	74.42	**75.42**
Cyclist AP	Easy	72.37	59.12	76.26	85.15	**85.92**	85.87	85.61
	Mod	63.36	68.00	76.71	84.70	85.64	86.10	**86.52**
(IoU = 0.5)	Hard	60.27	60.13	77.00	84.94	78.02	86.21	**86.21**
	Easy	68.11	61.84	75.99	81.54	80.61	85.03	**85.13**
mAP	Mod	62.22	63.41	77.72	80.39	81.46	83.09	**83.29**
	Hard	60.60	58.26	78.48	81.13	79.33	83.51	**83.85**
FPS		108	102	78	72	71	68	48

2D object detection results in the bird’s eye view perspective on the KITTI dataset. The evaluation metric is AP (Average Precision). The Intersection over Union (IoU) threshold is 0.7 for Car and 0.5 for Pedestrian/Cyclist. The bolded data are the highest scores for this item.

**TABLE 3:** Experimental results of the ablation experiments were used to verify the contribution of the improvements in this paper.

Improved part		Method A	Method B	Method C	Method D	Method E	Method F	Method G
E-RPN		√	√	√	√			
RepVGG-A2			√	√	√	√	√	√
FPN				√	√	√	√	√
Height detector					√	√	√	√
CBAM							√	√
Higher resolution								√
Car AP	Easy	67.72	43.88	49.68	92.50	87.37	**97.01**	96.56
	Mod	64.00	41.19	45.06	86.89	87.44	88.98	**89.10**
(IoU = 0.7)	Hard	63.01	37.68	43.24	87.51	87.90	89.26	**89.32**
Pedestrian AP	Easy	41.79	27.06	53.78	65.67	67.31	70.53	**71.33**
	Mod	39.70	28.62	55.99	68.11	69.92	72.53	**73.26**
(IoU = 0.5)	Hard	35.92	29.70	57.58	69.52	70.88	73.42	**74.11**
Cyclist AP	Easy	68.17	62.92	52.58	85.15	84.77	**85.81**	84.76
	Mod	58.32	69.83	48.34	84.70	76.79	**85.92**	85.60
(IoU = 0.5)	Hard	54.30	69.60	49.06	84.94	76.99	86.13	**86.72**
	Easy	59.22	44.62	52.01	81.11	79.82	**84.45**	84.21
mAP	Mod	54.00	46.55	49.80	79.90	78.05	82.48	**82.65**
	Hard	51.07	45.66	49.96	80.66	78.59	82.94	**83.38**
FPS		108	102	78	72	71	68	48

3D object detection results on the KITTI dataset. The evaluation metric is AP (Average Precision). The Intersection over Union (IoU) threshold is 0.7 for Car and 0.5 for Pedestrian/Cyclist. The bolded data are the highest scores for this item.

Method A is the original Complex-YOLO. It can be seen that the running speed is excellent and can reach 108 FPS on RTX3070Ti, but the detection accuracy is low.

Method B is a modified Complex-YOLO based RepVGG-A2 feature extraction network. Method B has higher detection accuracy for large objects like Car but lower detection accuracy for Pedestrian in 2D object detection in the bird’s-eye view perspective. The analysis found that the output feature map of RepVGG-A2 has a down-sampling multiplicity of 32, i.e., if the BEV-map with an input resolution of 608 × 608, then the output feature map size is 19 × 19. Such a smaller resolution means a larger perceptual field, which is not conducive to the prediction of small-sized objects. Method B has low accuracy in 3D object detection. In terms of detection speed, RepVGG still maintains a good detection speed, which can reach 102 FPS, because it consists of all 3 × 3 convolution in the inference stage and has no multi-branch structure.

Method C builds the FPN structure based on method B. Compared with method B, method C builds the FPN structure to accomplish the object detection of different sizes (38 × 38, 76 × 76, 152 × 152) at three different resolutions, greatly improving the 2D object detection accuracy of Pedestrian and Cyclist in the bird’s-eye view perspective, as well as the 3D object detection accuracy of Car and Pedestrian. In terms of detection speed, due to the construction of the FPN structure, which makes the network more complex, the detection speed is reduced to 78FPS, but the performance requirement of real-time detection is met.

Method D is based on method C, which adds the height detector. The addition of height detection in method D improves both 2D object detection accuracy in the bird’s-eye view perspective and 3D object detection accuracy.

Method E is based on method D, which removes the E-RPN structure and completes the detection of the orientation angle directly in the Cartesian coordinate system. The detection accuracy is also improved compared with method D.

Method F is an improved FPN structure built based on method E, the CBAM attention mechanism part is added to further improve the attention of the network for small objects.

Method G adds one more feature fusion operation of 2× up-sampling with adjacent resolution to the FPN structure based on method F, and complete the 3D object detection task at a higher resolution.

**FIGURE 9:**
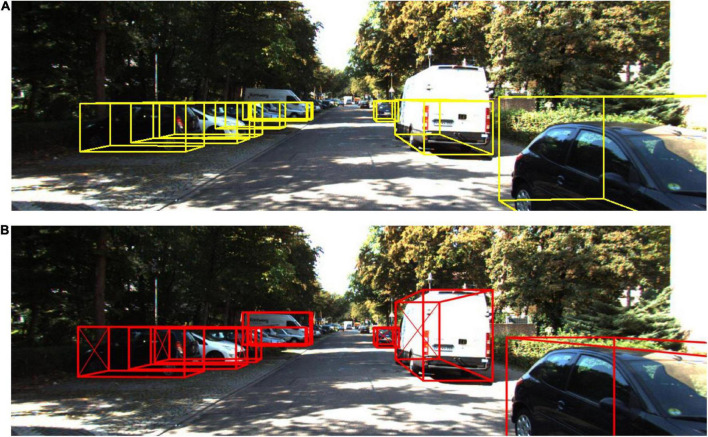
Comparison of our method with the detection results of Complex-YOLO. **(A)** 3D detection results of Complex-YOLO. **(B)** 3D detection results of our method.

To express the improvements in this paper more intuitively, the improved algorithm and the detection results of the original Complex-YOLO are converted into images for presentation. Figure 9 shows the detection results of the two algorithms. From Figure 9, it can be seen that the algorithm in this paper has a great improvement in 3D detection results compared to the original algorithm. Although the detection speed is slower than the original Complex-YOLO algorithm, it meets the performance requirements of real-time detection perfectly.

### 4.3. Comparative experiments and analysis with other methods

To further validate the advantages of the algorithms in this paper, we use the KITTI dataset as test data and compare it to other 3D object detection algorithms based on point cloud involved in this paper in three aspects: detection accuracy, detection speed, and memory usage. In addition, this paper also compares some subsequently improved algorithms of Complex-YOLO (Complex-YOLOv3 and Complex-YOLOv4; these algorithms all improve on Complex-YOLO according to the evolution of the subsequent YOLO series algorithms, but all maintain the original YOLO series network + E-RPN structure; Complex YOLOv3 with YOLOv3 + E-RPN and Complex YOLOv4 with YOLOv4 + E-RPN). We used the same data set division (6,000 data for training and 1,481 data for validation) for testing on GTX1060. Table 4 shows the 2D object detection results in the bird’s eye view. Table 5 shows the 3D object detection results. Table 6 shows the detection speed and memory usage for the same test conditions.

**TABLE 4:** Experimental results of comparison experiments with other algorithms.

Methods	Car AP (IoU = 0.7)			Pedestrian AP (IoU = 0.5)			Cyclist AP (IoU = 0.5)		
	Easy	Mod	Hard	Easy	Mod	Hard	Easy	Mod	Hard
PointRCNN (Shi et al., 2019)	90.64	89.54	88.68	86.94	**78.69**	74.70	**95.43**	**87.14**	**86.24**
PV-RCNN (Shi et al., 2020a)	99.14	89.91	89.57	73.85	71.56	69.60	91.70	82.88	81.73
SECOND (Yan et al., 2018)	98.01	88.98	87.72	67.49	65.59	62.75	90.89	84.26	83.52
PartA^2^ (Shi et al., 2020b)	98.81	89.52	89.09	76.71	70.14	68.65	94.03	83.94	83.33
PointPillars (Lang et al., 2019)	97.33	**90.04**	89.40	66.74	61.28	58.83	91.12	83.82	81.72
VoxelRCNN (Deng et al., 2021)	**99.21**	89.63	89.16	**75.01**	70.01	67.49	94.15	83.80	82.40
Complex-YOLOv3	71.73	74.87	78.73	54.74	59.44	62.39	64.06	74.23	67.71
Complex-YOLOv4	71.26	74.35	78.26	49.98	54.34	57.54	52.11	60.72	56.01
Our method	96.52	89.80	**89.92**	73.26	73.56	**75.42**	85.61	86.52	86.21

2D object detection results in the bird’s eye view perspective on the KITTI dataset. The evaluation metric is AP (Average Precision). The Intersection over Union (IoU) threshold is 0.7 for Car and 0.5 for Pedestrian/Cyclist. The bolded data are the highest scores for this item.

**TABLE 5:** Experimental results of comparison experiments with other algorithms.

Methods	Car AP (IoU = 0.7)			Pedestrian AP (IoU = 0.5)			Cyclist AP (IoU = 0.5)		
	Easy	Mod	Hard	Easy	Mod	Hard	Easy	Mod	Hard
PointRCNN	90.31	86.05	79.86	**86.26**	**77.97**	69.88	**95.44**	**87.15**	86.11
PV-RCNN	**98.69**	**89.23**	88.52	72.10	68.78	65.21	89.65	81.39	80.49
SECOND	98.01	88.97	87.72	65.80	63.49	60.05	90.89	84.26	83.52
PartA^2^	90.51	88.64	87.44	72.46	69.40	67.01	93.56	83.57	83.16
PointPillars	90.04	88.19	84.63	63.09	58.07	55.77	89.91	82.81	80.54
VoxelRCNN	90.22	88.41	87.06	74.95	67.11	65.40	94.02	83.68	82.30
Complex-YOLOv3	61.16	55.69	61.13	51.63	56.39	59.51	62.94	64.55	65.60
Complex-YOLOv4	62.39	56.61	61.81	47.94	51.82	55.17	51.78	52.90	54.35
Our method	96.56	89.10	**89.32**	71.33	73.26	**74.11**	84.76	85.60	**86.72**

3D object detection results on the KITTI dataset. Comparison of the results of 3D object detection on the KITTI dataset with other algorithms. The evaluation metric is AP (Average Precision). The Intersection over Union (IoU) threshold is 0.7 for Car and 0.5 for Pedestrian/Cyclist. The bolded data are the highest scores for this item.

**TABLE 6:** Experimental results of comparison experiments with other algorithms.

Methods	FPS	Memory
PointRCNN	3	1093Mib
PV-RCNN	3.8	1439Mib
SECOND	11	889Mib
PartA^2^	3	1021Mib
PointPillars	18	931Mib
VoxelRCNN	8	991Mib
Complex-YOLOv3	15	1195Mib
Complex-YOLOv4	12	1401Mib
Our method	20	841Mib

The detection speed and memory usage of each algorithm was compared on the GTX1060.

Tables 4, 5 show that Efficient Complex-YOLO has a high detection accuracy for tiny objects and objects with a high detection difficulty. Table 6 shows that Efficient Complex-YOLO performs well in terms of detection speed and memory usage in the same test environment.

## 5. Conclusion

In light of Complex-YOLO’s weaknesses in detection accuracy, this paper proposes a more efficient 3D object detection algorithm based on a series of improvements to Complex-YOLO. The RepVGG is used to increase the network’s depth and improve model fitting capabilities. To address the issue of poor detection accuracy of small objects in the original algorithm, improved FPN structure greatly improves the network’s accuracy for small objects. The Anchor-free + height detector method is used in the head network, and the original E-RPN structure is removed, which improves the network’s detection accuracy and compensates for the lack of height detection in the original algorithm. Although these improvements reduce the detection speed to some extent, the Efficient Complex-YOLO algorithm proposed in this paper can fully meet the performance requirements of real-time detection, reaching 48FPS on RTX3070Ti (it can guarantee a detection speed of 20FPS even on GTX1060). It has been experimentally proved that the method proposed in this paper has more advantages than Complex-YOLO. Compared with the subsequently improved algorithms of Complex-YOLO (Complex-YOLOv3 and Complex-YOLOv4), they all continue the original framework of Complex YOLO and do not achieve object height detection, so the proposed algorithms in this paper are more advantageous. The mAP of 2D object detection in bird’s eye view perspective is 85.13, 83.29, and 83.85 under Easy, Mod, and Hard conditions, respectively, while the mAP of 3D object detection is 84.21, 82.65, and 83.38 under Easy, Mod, and Hard conditions, respectively. Although Efficient Complex-YOLO falls short of top 3D object detection algorithms (e.g., PV-RCNN), it has a smaller memory usage and faster detection speed. In some applications with limited cost, it is still a method worth considering.

Point-based or Voxel-based 3D object detection algorithms, after the data pre-processing stage, often send a large amount of data into the network, which also tends to take a lot of arithmetic power consuming 3D convolution or fully connected layer for feature extraction, which causes the problem of slow detection speed and high memory occupation. The data processing method used in the Grid-based 3D object detection algorithm can significantly reduce the number of parameters input into the network and adopt the same structural form as the 2D object detection algorithm in the network, which can significantly improve the detection speed and reduce the memory occupation, and this paper also achieves good results in terms of detection speed and detection accuracy by improving Complex-YOLO. However, the data processing method like the one used in this paper will cause data loss in the encoding stage of the BEV-map, especially in the height information of the Z-axis direction, which largely limits the accuracy improvement of the algorithm. We suggest that the reader improve the data preprocessing or try other data processing methods to encode the point cloud to avoid the loss of information as much as possible.

## Data availability statement

The original contributions presented in this study are included in the article/supplementary material, further inquiries can be directed to the corresponding author.

## Author contributions

AT and TY: overall work direction. YS: algorithm design and essay writing. YS and ZS: code writing and software implementation. All authors have read and agreed to the published version of the manuscript.
